# Psychosocial Adjustment and Mental Distress Associated With In-Game Purchases Among Japanese Junior High School Students

**DOI:** 10.3389/fpsyg.2021.708801

**Published:** 2021-08-03

**Authors:** Hiroki Shinkawa, Tomonari Irie, Masanori Tanaka, Kengo Yokomitsu

**Affiliations:** ^1^Research Center for Child Mental Development, Graduate School of Medicine, Hirosaki University, Hirosaki, Japan; ^2^School of Education and Culture, Hokusho University, Ebetsu, Japan; ^3^Faculty of Business Administration, Hokkai-Gakuen University, Sapporo, Japan; ^4^Faculty of Health and Welfare, Kawasaki University of Medical Welfare, Kurashiki, Japan

**Keywords:** online gaming, in-game purchases, strengths and difficulties, depression, adolescents

## Abstract

In-game purchases, including microtransactions and loot box spending, are the monetization systems of free-to-play online games. Although some studies have suggested that excessive in-game purchases increase the risk of psychosocial maladjustment and mental distress as well as predict future problematic gaming and gambling practices, empirical studies on problematic behavioral patterns related to in-game purchasing among adolescents are lacking. This study sought to explore whether knowing the style of in-game purchases (non-purchase, planned purchase, or unplanned purchase) could be useful when characterizing maladaptive behavior among adolescents from the perspective of psychosocial adjustment and mental distress. A total of 335 junior high school students (aged 12–15 years) participated in the survey and completed a questionnaire assessing daily online gaming usage, in-game purchases, psychosocial adjustment, and mental distress. The results showed that (1) 30.7% of students had previously made in-game purchases, and at least 14.0% had made unplanned in-game purchases; (2) 19.2% of the users who had made unplanned purchases had spent greater than or equal to their actual monthly allowance within the past month, and (3) unplanned purchase gamers exhibited more behavioral problems and peer problems regarding psychosocial adjustment compared to planned purchase gamers, and more overall difficulties compared to non-purchasers. Meanwhile, more hyperactivity/inattention was seen among in-game purchasers compared to non-purchasers, regardless of whether the purchase was planned or unplanned. These findings support that understanding whether adolescents make unplanned in-game purchases could be a useful approach to describing the characteristics of online gamers with maladaptive tendencies.

## Introduction

Online gaming is one of the fastest growing industries in North America, Europe, and Asia. Its growth is especially rapid in Japan. Japanese users accounted for a 23% share ($1.45bn) of global mobile game revenue in 2019 (Sensor Tower Store Intelligence, 2020). In recent years, consumer behavior in online games has been supported by microtransactions, whereby small amounts of real-world money are spent to purchase virtual goods, new playable characters, or other advantages for sale in free-to-play games (King and Delfabbro, 2018). Loot boxes are another form of in-game purchases that provide players with a randomized reward of uncertain value (Zendle et al., 2020). The inclusion of in-game purchases in online games appears to be a successful strategy for motivating young players to spend money on what would otherwise be free. Indeed, research has shown that among Japanese teenagers and young adults, the longer the time they are exposed to limited-time loot boxes, the higher the rate of subsequent spending on mobile games (Shibuya et al., 2019).

Although Internet gaming might have some benefits, such as providing emotional excitement and increasing social connectedness, there are concerns about some structural similarities between in-game purchases (i.e., microtransaction spending and loot box engagement) and problem gambling among adolescents in terms of results uncertainty, the random chance involved, and the option of avoiding losses (Drummond and Sauer, 2018; Griffiths, 2019). Recent research has suggested that purchasing loot boxes in games is linked to problem gambling (Zendle and Cairns, 2019). Similar results were shown in a survey of adolescents aged 12–16 years (Kristiansen and Severin, 2020), and a stronger association was reported in a survey of adolescents aged 16–18 years (Zendle et al., 2019). In psychological terms, a preference for loot boxes was significantly associated with gambling-related cognitive distortions (Brooks and Clark, 2019). Furthermore, the physiological arousal patterns of gamers during microtransactions are similar to those of problematic gamblers (Brady and Prentice, 2019).

On the other hand, loot box purchases have also been found to be associated with the frequency at which adolescents play video games, and both of these factors have the potential to indirectly increase mental distress by increasing the risk of problematic video gaming (Li et al., 2019). However, while loot-box spending has shown mild associations with both negative mood and psychological distress, loot-box spending has also been associated with more positive moods in a cross-national survey (Drummond et al., 2020). Given that adolescents’ game playing is more vulnerable to external incentives such as media advertising than adults’ (Griffiths et al., 2004), regardless of whether the outcomes are positive or negative, in-game purchases could become even more prevalent among adolescents. Indeed, a cohort study among Japanese junior high school students reported that 3.5% of adolescents purchased loot boxes (Ide et al., 2021). Nevertheless, little is known about what individual behavioral patterns may increase the risk of psychosocial maladjustment and mental distress among adolescent in-game purchasers.

Some speculate that it would not be appropriate to uniformly consider game payment to be a problem because the adverse effects of in-game purchases vary according to the behavioral characteristics of the individual making the purchases. Prior studies highlight that online gaming motives (e.g., social, escape, and coping) play a role in determining whether a gamer will overspend while playing a game (Mäntymäki and Salo, 2015; Laconi et al., 2017). According to a theoretical model, excessive Internet game users tend to face difficulties in executive control and reward sensation and tend to choose immediate and short-term benefits (Dong and Potenza, 2014). Another study has shown that difficulties with emotional regulation lead to problematic smartphone use among adolescents (Fu et al., 2020). Although the current criteria for gaming disorders do not refer to the escalating loss of real money, some problematic game players may spend more than they can afford on in-game purchases (King et al., 2019).

Such problematic in-game purchasing in adolescents could be viewed as typical of risk behavior in this age group owing to their immature neurological development and difficulties with behavioral inhibition (e.g., Chambers et al., 2003). Reyna and Farley (2006) suggested that there are two patterns of risk behavior in adolescents: planned and unplanned. In this framework, planned risk behavior is referred to as rational risk, which describes behavior resulting from a benefit-oriented decision made when comparing the behavior’s potential benefits and risks. Unplanned risk behavior is referred to as reactive risk, which is unplanned behavior made in response to environmental stimuli or internal experiences. Compared to those who engage in planned risk behaviors, adolescents who engage in unplanned risk behaviors are more likely to choose high-risk behaviors in tasks that measure gambling-like risk preferences (Maslowsky et al., 2011). Thus, when considering in-game purchases as part of a tendency toward risk behavior, knowing whether their purchasing is planned or unplanned may offer important insights into what constitutes healthy gameplay in adolescents. In other words, unplanned in-game purchases, which are not based on rationality and are incurred by internal or external experience without prior planning, are considered to lead to more maladaptive online game playing.

Understanding the behavior of adolescents engaged in in-game purchases is an important basis for developing policies and interventions to prevent or mitigate the risks associated with adolescent problematic online gaming. In particular, earlier studies have pointed out that risk behavior in junior high school years may be indicative of an overall vulnerability to developing a gambling problem (Castrén et al., 2015). The present study sought to describe the features of psychosocial adjustment and mental distress associated with in-game purchases among early adolescents, using an exploratory approach. Our specific research questions were as follows: (1) What percentage of junior high school students are involved in in-game purchasing? (2) How much and under what circumstances do they make unplanned in-game purchases? (3) To what extent are planned or unplanned in-game purchases associated with psychosocial adjustment and mental distress?

## Materials and Methods

### Ethical Considerations

The protocol of the current study was approved by the Ethics Committee of Hokusho University (No. 2020-019). Regarding the protection of personal data, this study adhered to the Board of Education’s information security policies.

### Participants

The schools that participated in this study were included in an annual survey conducted by the Research Center for Child Mental Development in collaboration with local boards of education to detect the mental health needs of junior high school students. A total of 344 Japanese students from two junior high schools were surveyed in July 2020. These schools are located in a small city in Japan with a population of less than 20,000 people. In the surveyed area, no COVID-19 infections were reported before October 2020, the impact of the COVID-19 pandemic had been minimal, and the classes had returned to normal since the state of emergency was lifted in May 2020. The response rate was 97.4% (*N* = 335/344). The sample without missing values for the measures included 335 participants aged 12–15 years (100 seventh graders, 138 eighth graders, and 97 ninth graders), comprised of 173 boys and 162 girls.

### Measures

#### Daily Online Gaming

The participants were asked how many hours per day (weekday or weekend) they played online games. They were also asked to choose the time zone they played in most frequently, both on weekdays and weekends. The answer choices of the time zone ranged from six options (i.e., 0:00–4:00, 4:00–8:00, 8:00–12:00, 12:00–16:00, 16:00–20:00, and 20:00–0:00).

#### In-Game Purchases

We asked all participants whether they had made any in-game purchases during the past month. Furthermore, those who had made in-game purchases were also asked about the number of in-game purchases they had made and whether they had made any unplanned in-game purchases. In this context, unplanned in-game purchases were defined as “spending money that had not been planned for spending due to impatience (e.g., to advance in the game, to get a rare item or character they wanted, etc.) in online games.” Referring to this definition, participants were asked whether they had ever made unplanned in-game purchases and, if so, how many unplanned purchases they had made in the past month. Participants were informed that purchases of multiple items during one instance of gameplay was considered to be one unplanned purchase. Since the form of a microtransaction depends on the online game’s system and is therefore fluid, participants were not asked to make further distinctions. All participants were also asked on an open-ended question about the amount of monthly allowance they were able to use freely. We asked the following questions to only unplanned purchase users: (1) What was the number of unplanned purchases they had made? (2) What were the titles of the games in which they had made unplanned purchases? and (3) What were the motivation and reasons of unplanned purchases?

#### Psychosocial Adjustment

Psychosocial adjustment was evaluated using the Strengths and Difficulties Questionnaire (SDQ; Goodman, 1997). The SDQ is a 25-item self-report questionnaire that assesses a broad range of emotional and behavioral problems in school-aged children. The scale is comprised of one subscale for strengths (prosocial behavior) and four subscales for difficulties (emotional symptoms, conduct problems, hyperactivity/inattention, and peer problems), each containing five items. The total difficulties score is calculated as the sum of the four subscales for difficulties. Each item was rated on a 3-point Likert scale (0 = *not true*, 1 = *somewhat true*, or 2 = *certainly true*). The total difficulty scores ranged from 0 to 40, with higher scores indicating greater challenges. The SDQ has been translated into Japanese and its reliability and validity have been verified in Japan (Noda et al., 2013). Cronbach’s α coefficients for the SDQ were as follows: prosocial behavior (α = 0.62), emotional symptoms (α = 0.71), conduct problems (α = 0.39), hyperactivity/inattention (α = 0.62), peer problems (α = 0.49), and total difficulties (α = 0.76) in Japanese community-based samples (Noda et al., 2013); prosocial behavior (α = 0.72), emotional symptoms (α = 0.67), conduct problems (α = 0.42), hyperactivity/inattention (α = 0.67), peer problems (α = 0.48), and total difficulties (α = 0.81) in the current samples.

#### Mental Distress

Mental distress was assessed using the Patient Health Questionnaire for Adolescents (PHQ-A; Johnson et al., 2002). The PHQ-A is widely used as a brief screening measure for major depression in adolescents. The scale items consist of nine items based on episodes in the diagnostic criteria for depression. Students rated each item on a 4-point Likert scale (0 = *not at all* to 3 = *nearly every day*). The total scores ranged from 0 to 27, with higher values representing more severe levels of distress. The distribution patterns of the PHQ-A among Japanese adolescents have been previously reported (Adachi et al., 2020). Cronbach’s α coefficient for the Japanese version of the PHQ-A has not been previously reported; however, it was sufficient (α = 0.83) in the current samples.

### Procedures

Using the board of education as an intermediary, an outline of the investigation was given in writing to the school principals, and consent to participate in the study was obtained in advance. Before the survey was conducted, the study’s purpose, significance, and methodology were explained in writing to students and their parents or legal guardians. Homeroom teachers distributed the questionnaire in each classroom according to the survey manual, and questionnaires were only given to students after obtaining parental consent. Students were considered to obtain consent by submitting the questionnaire. The survey was conducted offline.

### Statistical Analyses

Descriptive statistics were calculated for the total sample and among those who made unplanned purchases. A primary analysis was performed using an analysis of variance (ANOVA) to reveal the main effects of in-game purchase styles (i.e., non-purchase, planned purchase, unplanned purchase) on each measure. Multiple comparisons were conducted using Shaffer’s *post hoc* test. Statistical significance was set at *p* < 0.05. Partial η^2^ and Cohen’s *d* were adopted for the evaluation of effect size. Missing values were handled using pairwise deletion. All statistical analyses were conducted using R version 4.0.4 (R Core Team, 2021).

## Results

### Demographic Characteristics

Descriptive statistics, including the participants’ characteristics and information related to online gaming, such as time spent gaming, time zone of playing games, style of in-game purchases, and monthly allowance are presented in Table 1. Participants were asked their time spent gaming and monthly allowance on an open-ended question; however, for convenience, the answers were organized into categories. Most participants played online games for more than 1 h per weekday (70.1%) and per weekend (83.9%), and a relatively large number of participants spent between 1 and less than 2 h gaming on weekdays (37.0%) and 2 and less than 3 h gaming on weekends (22.4%). The time of day during which games were playing most on weekdays was after 20:00 (53.1%), for more than half of participants, while on weekends the time of day varied between 12:00 and 0:00. Further, 30.7% of participants had already made in-game purchases, and at least 14.0% had made unplanned in-game purchases. The modal value of participants’ monthly allowances was in the 3,000 JPY range, but the average value was 2,347 JPY.

**TABLE 1:** Demographic characteristics (*N* = 335).

Variable	Category	*n* (%)
Time spent on online gaming (per weekday)	less than 1 h	92 (27.5%)
	1 to less than 2 h	124 (37.0%)
	2 to less than 3 h	66 (19.7%)
	3 to less than 4 h	22 (6.6%)
	4 to less than 5 h	10 (3.0%)
	5 to less than 6 h	5 (1.5%)
	6 h or more	10 (3.0%)
Time spent on online gaming (per weekend)	less than 1 h	47 (14.0%)
	1 to less than 2 h	42 (12.5%)
	2 to less than 3 h	75 (22.4%)
	3 to less than 4 h	60 (17.9%)
	4 to less than 5 h	35 (10.4%)
	5 to less than 6 h	24 (7.2%)
	6 h or more	45 (13.4%)
Time zone on online gaming (per weekday)	4:00–8:00	8 (2.3%)
	8:00–12:00	6 (1.7%)
	12:00–16:00	1 (0.3%)
	16:00–20:00	59 (17.2%)
	20:00–0:00	121 (53.1%)
	0:00–4:00	13 (3.8%)
Time zone on online gaming (per weekend)	4:00–8:00	11 (3.3%)
	8:00–12:00	38 (11.3%)
	12:00–16:00	79 (23.4%)
	16:00–20:00	53 (15.8%)
	20:00–0:00	54 (16.1%)
	0:00–4:00	9 (2.7%)
The style of in-game purchases	Non-purchase users	232 (69.3%)
	Planned purchase users	56 (16.7%)
	Unplanned purchase users	47 (14.0%)
Monthly allowance	Less than 1,000 JPY	15 (4.5%)
	1,000–1,999 JPY	84 (25.1%)
	2,000–2,999 JPY	66 (19.7%)
	3,000–3,999 JPY	109 (32.5%)
	4,000 JPY or more	34 (10.1%)

*JPY = Japanese Yen (1,000 JPY = 9.12 USD).*

Of the 47 participants (14.0%) who had made unplanned in-game purchases, about half (51.1%) had engaged in unplanned purchases within the past month (Table 2). Among those who had made unplanned purchases, 23.4% spent more than their average monthly allowance (2,347 JPY). Those who made purchases of more than 3,000 JPY (19.2%) spent greater than or equal to their actual monthly allowance. In contrast, individuals who spent less than 2,000 JPY (17.0%) did not spend more than their allowance. In terms of the type of game, *Fortnite* (51.1%) was the game in which the most in-game purchases were made and *Knives Out* (34.0%) was the second. Paying up in either of these games was common among participants who made unplanned purchases. The most common motivation for in-game purchases was obtaining rare items or characters (53.2%). Limited-time products were most often cited as reasons for unplanned purchasing (51.1%).

**TABLE 2:** Unplanned purchase user profiles (*n* = 47).

Variable	Category		*n* (%)
Number of unplanned purchases in the past month	0		23 (48.9%)
	1		17 (36.2%)
	2		3 (6.4%)
	3 or more		4 (8.5%)
Amount of unplanned purchases in the past month	0 JPY		23 (48.9%)
	less than 1,000 JPY		3 (6.4%)
	1,000–1,999 JPY		7 (14.9%)
	2,000–2,999 JPY		0 (0.0%)
	3,000–3,999 JPY		5 (10.6%)
	4,000 JPY or more		6 (12.8%)
Ratio of spending to actual monthly allowance for purchasers	Spending below 2,000 JPY		
		Less than their allowance	8 (17.0%)
		Equal to their allowance	0 (0.0%)
		More than their allowance	0 (0.0%)
	Spending of 3,000 JPY or more		
		Less than their allowance	0 (0.0%)
		Equal to their allowance	2 (4.3%)
		More than their allowance	7 (14.9%)
Game titles with purchases^*a*^	*Fortnite*		24 (51.1%)
	*Knives Out*		16 (34.0%)
	*Super Smash Bros.*		5 (10.6%)
	*Apex Legends*		4 (8.5%)
	*Yo-kai Watch Puni Puni*		4 (8.5%)
	*Monster Strike*		3 (6.4%)
	*Tsum Tsum*		3 (6.4%)
	Other titles		20 (42.6%)
Motivation for unplanned purchases^*a*^	Obtaining rare items or characters		25 (53.2%)
	Opening loot boxes		96 (12.8%)
	Progressing the game task		5 (10.6%)
	Competing with other players		3 (6.4%)
	Other purposes		2 (4.3%)
Reasons at the time of unplanned purchases^*a*^	Limited-time products		24 (51.1%)
	Plenty of pocket money		14 (29.8%)
	Limited-time discounts		9 (19.1%)
	Close to achieving my goal		2 (4.3%)
	Feeling stressed out		2 (4.3%)
	Invitation from friends		1 (2.1%)
	Other situations		6 (12.8%)

*JPY = Japanese Yen (1,000 JPY = 9.12 USD).*

*^a^Multiple answers allowed.*

### Comparisons of User Profiles, SDQ, and PHQ-A According to the Styles of In-Game Purchases

The results of the ANOVA showed the main effect of the styles of in-game purchases on time spent online gaming, SDQ subscale and total scores, and PHQ-A scores (Table 3). There was no significant difference in monthly allowance among the three groups. After multiple comparisons, time spent per weekend (*d* = 0.72), conduct problems (*d* = 0.35), and hyperactivity/inattention (*d* = 0.34) were observed to be significantly higher (adjusted *p* < 0.05) among planned purchasers compared to non-purchasers who reported lower time spent per weekend (*d* = 0.76), greater conduct problems (*d* = 0.74), higher hyperactivity/inattention (*d* = 0.34), more peer problems (*d* = 0.43), and greater total difficulties (*d* = 0.52). In addition, unplanned purchasers recorded a significantly higher (adjusted *p* < 0.05) amount of conduct problems (*d* = 0.40) and peer problems (*d* = 0.35) compared to planned purchasers. For time spent per weekday and PHQ-A, significant differences were not detected among the three groups after adjustment for multiple comparison.

**TABLE 3:** Comparison of user profiles, SDQ, and PHQ-A according to the styles of in-game purchase.

		Total (*N* = 335)		Non-purchase users (*n* = 232)		Planned purchase users (*n* = 56)		Unplanned purchase users (*n* = 47)		*F*	*Partial* η^2^	Shaffer’s *post hoc* test (*p* < 0.05)
		*M*	*SD*	*M*	*SD*	*M*	*SD*	*M*	*SD*			
**Online gaming**												
	Time spent (per weekday)	1.60	2.36	1.41	2.59	1.88	1.50	2.29	1.90	3.18*	0.02	
	Time spent (per weekend)	3.29	3.07	2.65	2.45	4.75	3.60	4.87	3.92	19.19**	0.11	Non < planned, unplanned
**Monthly allowance (JPY)**		2,347	1,242	2,319	1,277	2,390	977	2,400	1,370	0.11	<0.01	
**SDQ**												
	Prosocial behavior	6.83	2.15	6.92	2.14	6.52	2.28	6.77	2.09	0.60	<0.01	
	Emotional symptoms	2.98	2.58	2.92	2.60	3.04	2.59	3.07	2.46	0.05	<0.01	
	Conduct problems	2.01	1.54	1.79	1.24	2.29	1.78	2.89	2.12	11.18**	0.07	Non < planned < unplanned
	Hyperactivity/inattention	2.78	2.11	2.53	2.01	3.24	2.28	3.57	2.13	6.04**	0.04	Non < planned, unplanned
	Peer problems	2.11	1.58	2.00	1.57	2.09	1.55	2.72	1.60	3.38*	0.02	Non, planned < unplanned
	Total difficulties	9.88	5.74	9.23	5.36	10.69	5.98	12.19	6.85	5.54**	0.03	Non < unplanned
**PHQ-A**		4.71	4.76	4.19	4.29	5.61	4.98	5.89	6.00	3.89*	0.03	

*p < 0.05, **p < 0.01, JPY = Japanese Yen (1,000 JPY = 9.12 USD).

*SDQ = Strengths and Difficulties Questionnaire; PHQ-A = Patient Health Questionnaire for Adolescents.*

## Discussion

We sought to explore whether the style of in-game purchases made by adolescents could offer useful insights when characterizing those with maladaptive behavior from the perspective of psychosocial adjustment and mental distress by making three specific research questions.

*(1) What percentage of junior high school students are involved in in-game purchasing?*

The descriptive statistics showed that the majority of junior high school students, at least 70.1% on weekdays and 83.9% on weekends, played online games for more than one h, while 30.7% of these gamers made in-game purchases. Given the finding that only 3.5% of junior high school students in Japan purchase loot boxes (Ide et al., 2021), it is likely that microtransactions are a more accessible monetization system for adolescents than loot-box spending. Moreover, as our results indicate, there are not many excessive gamers or gamers who play more than 5 h per day regardless of whether it is a weekday (4.5%) or weekend (20.6%); thus, we can infer that in-game purchases also generally occur among recreational users. These results support the greater accessibility of in-game purchases provided by the microtransaction platform, which may be a factor contributing to the increased numbers of in-game purchases made by adolescents (e.g., King and Delfabbro, 2018).

*(2) How much and under what circumstances do they make unplanned in-game purchases?*

Slightly over half (51.1%) of those who made unplanned in-game purchases had engaged in purchasing in the past month, nearly one-fourth (23.4%) had spent more than the average monthly allowance of a junior high school student, and nearly one-fifth (19.2%) had spent equal or more than their actual monthly allowance on in-game purchases. While these financial losses may not be comparable to those of problematic gamblers, those could represent an excessive expenditure in the context of the ratio between the money spent and their allowance. Furthermore, Griffiths et al. (2004) noted that, in most cases where adolescent gamers made unplanned purchases, there were external incentives which may have had an impact, such as obtaining rare items and limited-time events. For example, *Fortnite* is a game in which in-game purchasing is common. It is a ”battle royale” online game (i.e., a last-man-standing, shooter-style game) that had more than 350 million registered users as of May 2020 (Statistica, 2021). A survey of adult *Fortnite* players showed that peers’ purchasing behaviors were one of the predictors of individual microtransaction spending (King et al., 2020), and it is possible that the purchasing of limited-time costumes called ”skins” is also receiving social reinforcement among the adolescent peers participating in this study. Compared with previous studies (Ide et al., 2021), the relatively small percentage (12.8%) of participants who made in-game purchases through opening loot boxes may merely reflect the low prevalence within microtransaction system of current popular games. In order to distinguish these spending ratios from typical adolescent trends these days, it is necessary to clarify normative data on the breakdown of adolescent expenditures in a larger sample, which includes planned in-game purchase users.

*(3) To what extent are planned or unplanned in-game purchases associated with psychosocial adjustment and mental distress?*

The outcome measures of time spent playing online games, psychosocial adjustment, and mental distress were compared among non-purchase gamers, planned purchase gamers, and unplanned purchase gamers. The results of the ANOVA showed that gamers who made in-game purchases (both planned and unplanned) spent significantly more time playing online games on weekends and had higher conduct problems and hyperactivity/inattention measured by the SDQ representing psychosocial maladjustment, compared with non-purchase gamers. A higher level of behavioral problems is a common risk factor for pathological gaming (Dong and Potenza, 2014). In addition, the higher levels of hyperactivity/inattention among in-game purchasers may be confounded by reward sensitivity, which is another common risk factor for problematic gambling (e.g., Kim et al., 2017). Overall, the results for total difficulties as measured by the SDQ were significant only for unplanned purchasers compared to non-purchasers, suggesting that while it is inappropriate to simply consider in-game purchases to be problematic, knowing the style of in-game purchases could be useful when describing the characteristics of online gamers with maladaptive tendencies on psychosocial outcomes.

More notably, the finding that conduct problems and peer problems were greater among unplanned purchase gamers than among planned purchase gamers. While the effect was minimal, this may suggest that unplanned purchase gamers are currently experiencing a lack of social approval in their offline life. Pathological gamers are likely to experience increased loneliness (Wang et al., 2019), and may spend more time and money on online games in order to increase the value of social acceptance obtained from online gaming (King and Delfabbro, 2014). It would be particularly important to support such adolescents not only in terms of the magnitude of financial loss, but also in terms of developing self-control and social networks online or offline.

On the other hand, the subscales of prosocial skills and emotional symptoms found in the SDQ and PHQ-A did not differ significantly among the in-game purchasing styles, which suggests that these are not shared characteristics of in-game purchasers. Previous studies have also shown little direct association between loot-box purchasing and mental distress (Li et al., 2019; Drummond et al., 2020; Hall et al., 2021). This wide variation in the comorbidity between pathological gaming and psychosocial problems was also observed in a meta-analysis by Ferguson et al. (2011). Given the controversy over the diagnostic criteria for gaming disorder (e.g., van Rooij et al., 2018), it would be inappropriate to conclude that in-game purchases have a uniformly negative effect on adolescents’ psychological adjustment. The results of this study instead suggest the view that in-game purchasing is not a factor that immediately hinders mental health promotion or social development among students, even if it is an unplanned behavior. To promote further discussion, it may be beneficial to identify the social context in which individuals adaptively engage in gaming with in-game purchases.

In this study, we attempted to describe the features of adolescents who made in-game purchases by classifying them as planned or unplanned purchasers based on the framework of their decision making (Reyna and Farley, 2006). Indeed, not all the unplanned in-game purchasing gamers were excessive spending, nor were they consistently spending every month. Hence, it should be noted that the distinction may oversimplify the subtype of in-game purchasing behavior. However, the findings of our research are meaningful in suggesting that the styles of in-game purchases may partially explain the differences in psychosocial adjustment among adolescents. The assumption of such distinctions could contribute to somewhat fairer discussion than immediately considering adolescents’ in-game purchases as problematic. Since the present study is a descriptive approach, we did not focus on the mechanism of unplanned in-game purchases, whereas previous studies showed that sensitivity seeking, working memory, and future orientation were associated with unplanned risk behaviors (Maslowsky et al., 2019). If these characteristics are also observed in unplanned purchasers of adolescents, it might be possible to predict the risk of unplanned spending. We argue that understanding whether adolescents make unplanned in-game purchases could be a useful approach to describing the characteristics of online gamers with maladaptive tendencies.

### Limitations and Future Directions

There are some limitations of this study that should be discussed. First, this study was based on a self-reported questionnaire survey. Thus, the effects of recall bias and social desirability are unavoidable. Therefore, it would be desirable to analyze smartphone activity information with the help of game developers in future studies (King et al., 2020). In addition, due to the low reliability of certain subscales within the SDQ, the robustness of the results remains questionable. It is recommended that the psychosocial outcomes are examined in light of ratings provided by a parent and/or teacher as well as the adolescents themselves. Second, since our subjects were junior high school students who lived in a specific area of Japan, there are limitations to the extent to which our findings can be applied to older adolescents or schools in different localities. A larger sample will be needed to determine the impact of planned and unplanned in-game purchases among adolescents. Third, although the survey was conducted in an area where the impact of the COVID-19 pandemic was minimal, given the reported differences in the effect sizes of the association between loot box spending and problematic gambling due to self-isolation status (Hall et al., 2021), generalizations of findings reported during the COVID-19 pandemic must be made with caution. Finally, this was a cross-sectional study. Therefore, it is not possible to claim a causal relationship between planned or unplanned in-game purchases and mental health outcomes. An alternative interpretation of the association between in-game purchases and worsening mental health among gamers could be that individuals with mental health problems may engage in gameplay with in-game purchases as a coping behavior to relieve their depressive symptoms. Future studies must examine the relationship between these variables, including the severity of online gaming, by using a longitudinal research design.

## Data Availability Statement

The raw data supporting the conclusions of this article will be made available by the authors, without undue reservation.

## Ethics Statement

The studies involving human participants were reviewed and approved by Ethics Committee of Hokusho University. Written informed consent to participate in this study was provided by the participants’ legal guardian/next of kin.

## Author Contributions

HS, TI, MT, and KY: study planning. HS: data collection, data analysis, and writing the manuscript. TI, MT, and KY: review and editing. All authors contributed to the article and approved the submitted version.

## Conflict of Interest

The authors declare that the research was conducted in the absence of any commercial or financial relationships that could be construed as a potential conflict of interest.

## Publisher’s Note

All claims expressed in this article are solely those of the authors and do not necessarily represent those of their affiliated organizations, or those of the publisher, the editors and the reviewers. Any product that may be evaluated in this article, or claim that may be made by its manufacturer, is not guaranteed or endorsed by the publisher.
